# Serous papillary adenocarcinoma possibly related to the presence of primitive oocyte-like cells in the adult ovarian surface epithelium: a case report

**DOI:** 10.1186/1757-2215-4-13

**Published:** 2011-08-09

**Authors:** Irma Virant-Klun, Thomas Skutella, Branko Cvjeticanin, Martin Stimpfel, Jasna Sinkovec

**Affiliations:** 1 Department of Obstetrics and Gynecology, University Medical Centre Ljubljana, Slovenia; 2 Institute for Anatomy and Cell Biology, Faculty of Medicine, University of Heidelberg, Heidelberg, Germany

**Keywords:** human, oocytes, ovarian surface epithelium, serous adenocarcinoma

## Abstract

**Introduction:**

The presence of oocytes in the ovarian surface epithelium has already been confirmed in the fetal ovaries. We report the presence of SSEA-4, SOX-2, VASA and ZP2-positive primitive oocyte-like cells in the adult ovarian surface epithelium of a patient with serous papillary adenocarcinoma.

**Case presentation:**

Ovarian tissue was surgically retrieved from a 67-year old patient. Histological analysis revealed serous papillary adenocarcinoma. A proportion of ovarian cortex sections was deparaffinized and immunohistochemically stained for the expression of markers of pluripotency SSEA-4 and SOX-2 and oocyte-specific markers VASA and ZP2. The analysis confirmed the presence of round, SSEA-4, SOX-2, VASA and ZP2-positive primitive oocyte-like cells in the ovarian surface epithelium. These cells were possibly related to the necrotic malignant tissue.

**Conclusion:**

Primitive oocyte-like cells present in the adult ovarian surface epithelium persisting probably from the fetal period of life or developed from putative stem cells are a pathological condition which is not observed in healthy adult ovaries, and might be related to serous papillary adenocarcinoma manifestation in the adult ovarian surface epithelium. This observation needs attention to be further investigated.

## Introduction

In fetal ovaries, the number of germ cells reaches a peak of ~6 to 7 million during the fifth month post-fertilization [1], after which germ cell reduction occurs during prenatal development, resulting in the presence of only 1 million of female germ cells before birth. Two mechanisms have been proposed to restrict the pool of female gametes during prenatal life: 1) germ cell degeneration inside the developing ovary and, 2) germ cell extension into the ovarian surface epithelium and exfoliation from the ovarian surface into the coelomic cavity [1-7]. Therefore, primitive oocytes can be found in the ovarian surface epithelium and on the surface of fetal ovaries, as revealed by the transmission and scanning electron microscopy. Germ cells may reach the site of ovarian surface epithelium by still retained amoeboid movements in early developmental stages, or are passively pushed there by the morphogenetic rearrangement of somatic cells in later stages of ovarian development. This phenomenon is observed in human fetal ovaries, might persist until the puberty, but is not present in adult ovaries. It has already been proposed that these residual primitive oocytes in the adult ovarian surface epithelium may give rise to abnormal cell growth, such as teratomas [1,2], but not much experimental evidence has been available.

Recent findings have confirmed the presence of putative stem cells in the adult human ovarian surface epithelium [8-10]; they can also be found in the ovaries of older women [9,11]. Putative stem cells are small round cells with diameters of 2 to 4 μm that express some markers of pluripotent stem cells and can develop *in vitro *into primitive oocyte-like cells. Putative stem cells found in adult ovarian surface epithelium resemble very small embryonic-like stem cells (VSELs) found in other adult tissues and organs [12-14]. It is proposed that VSELs originate in the epiblast and persist in adult tissues and organs from the embryonic period of life [15].

## Case report

**Figure 1:**
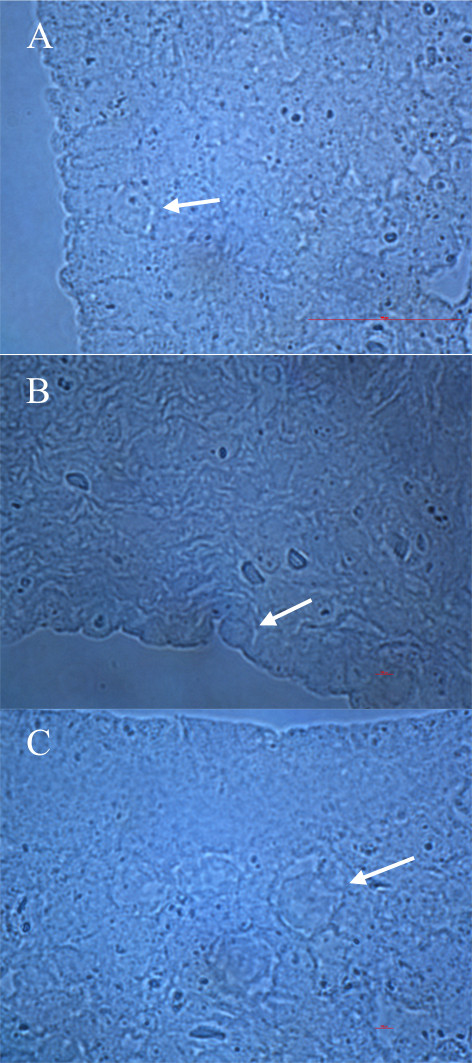
**Primitive oocytes (arrows) in the ovaries of a 15 week-old fetus histologically analyzed at our department**. **(A, B) **In the ovarian surface epithelium (light microscopy, magnification 1000×). **(C) **Just below the ovarian surface epithelium (light microscopy, magnification 1000×).

**Figure 2:**
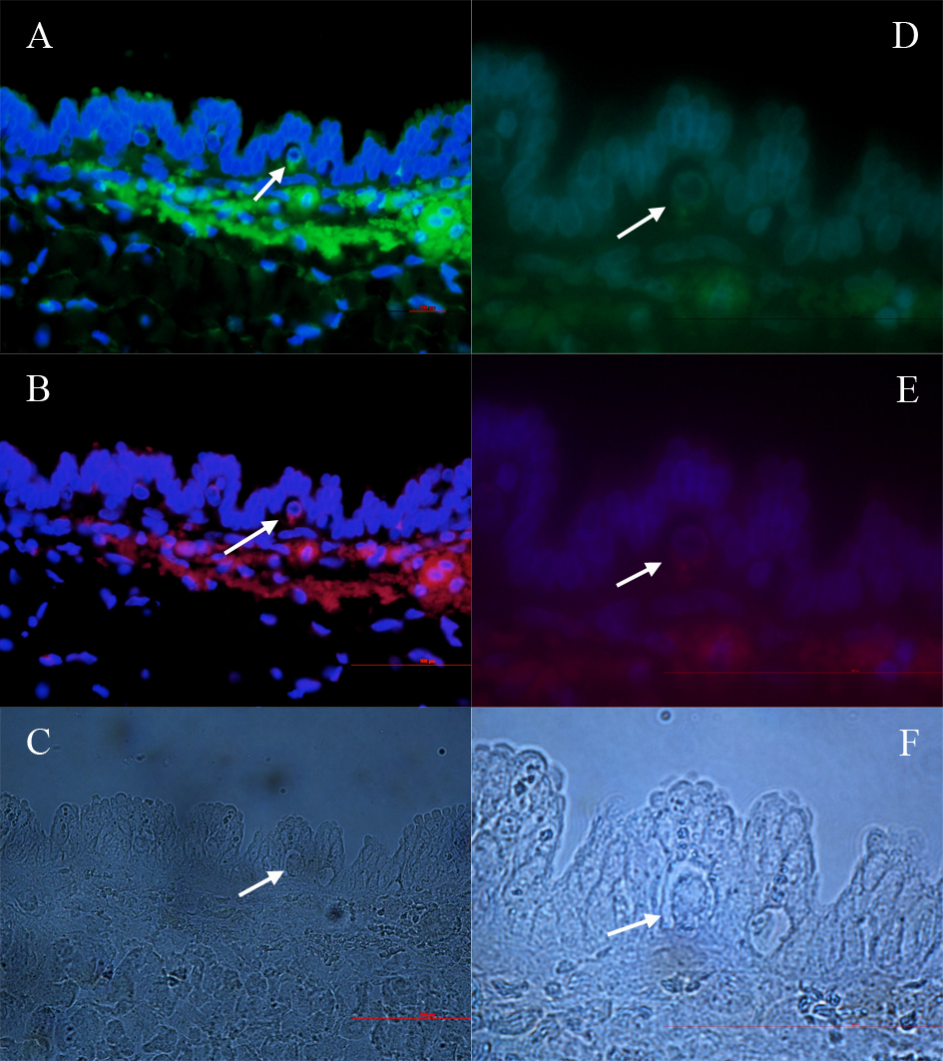
**Primitive oocyte-like cells (arrows) in the ovarian surface epithelium above the autofluorescent necrotic malignant tissue of a patient with serous papillary adenocarcinoma**. **(A, D) **SSEA-4-positive cells (green). **(B, E) **SOX-2-positive cells (red). **(C, F) **Non-stained cells. (A, B, C: fluorescent and light microscopy, magnifications 400×; D, E, F: fluorescence and light microscopy, magnifications 1000×).

**Figure 3:**
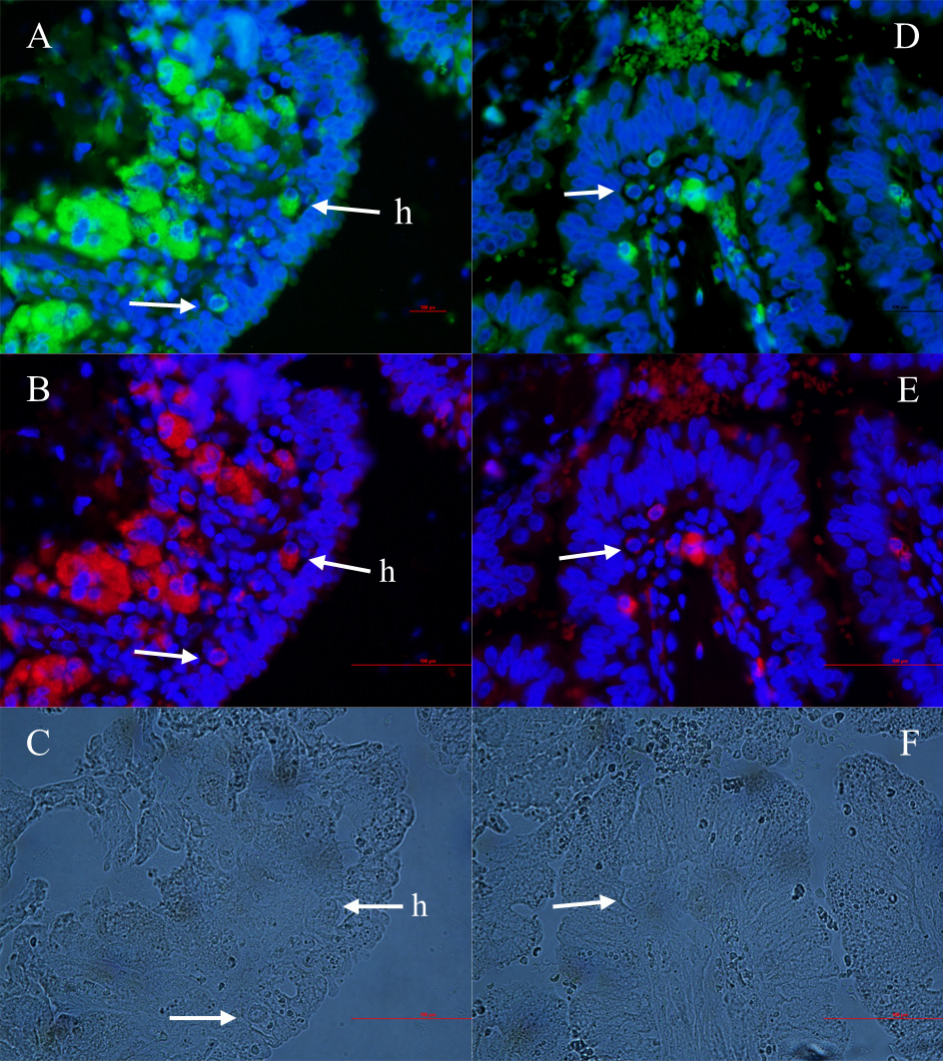
**Normal and hypertrophic primitive oocyte-like cells (arrows) in the ovarian surface epithelium above the autofluorescent necrotic malignant tissue of a patient with serous papillary adenocarcinoma**. **(A, D) **SSEA-4-positive cells (green). **(B, E) **SOX-2-positive cells (red). **(C, F) **Non-stained cells. (fluorescence and light microscopy, magnifications 400×). *Legend*: h-hypertrophic/necrotic cells.

**Figure 4:**
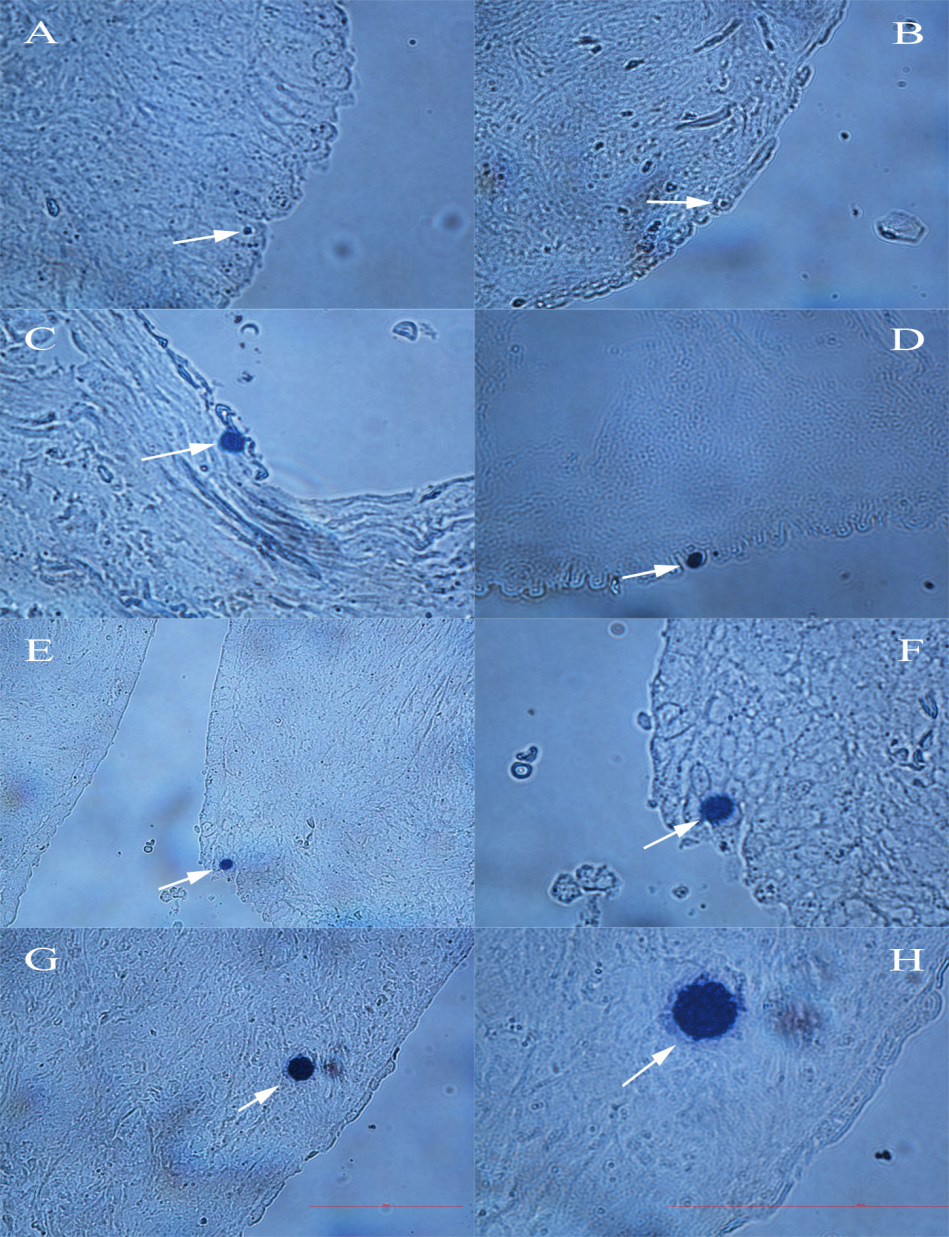
**VASA-positive cells (arrows) in the ovarian surface epithelium and cortex**. **(A, B) **Smaller cells with diameters of 2 to 4 μm in the epithelium. **(C-F) **Bigger cells with diameters of 10 μm in the epithelium. **(G, H) **Rare cells with diameters of approximately 30 μm in the cortex. (light microscopy, magnifications 400× and 1000×). *Legend*: blue-positive cells (Peroxidase/True Blue). Scale bar: 100 μm.

**Figure 5:**
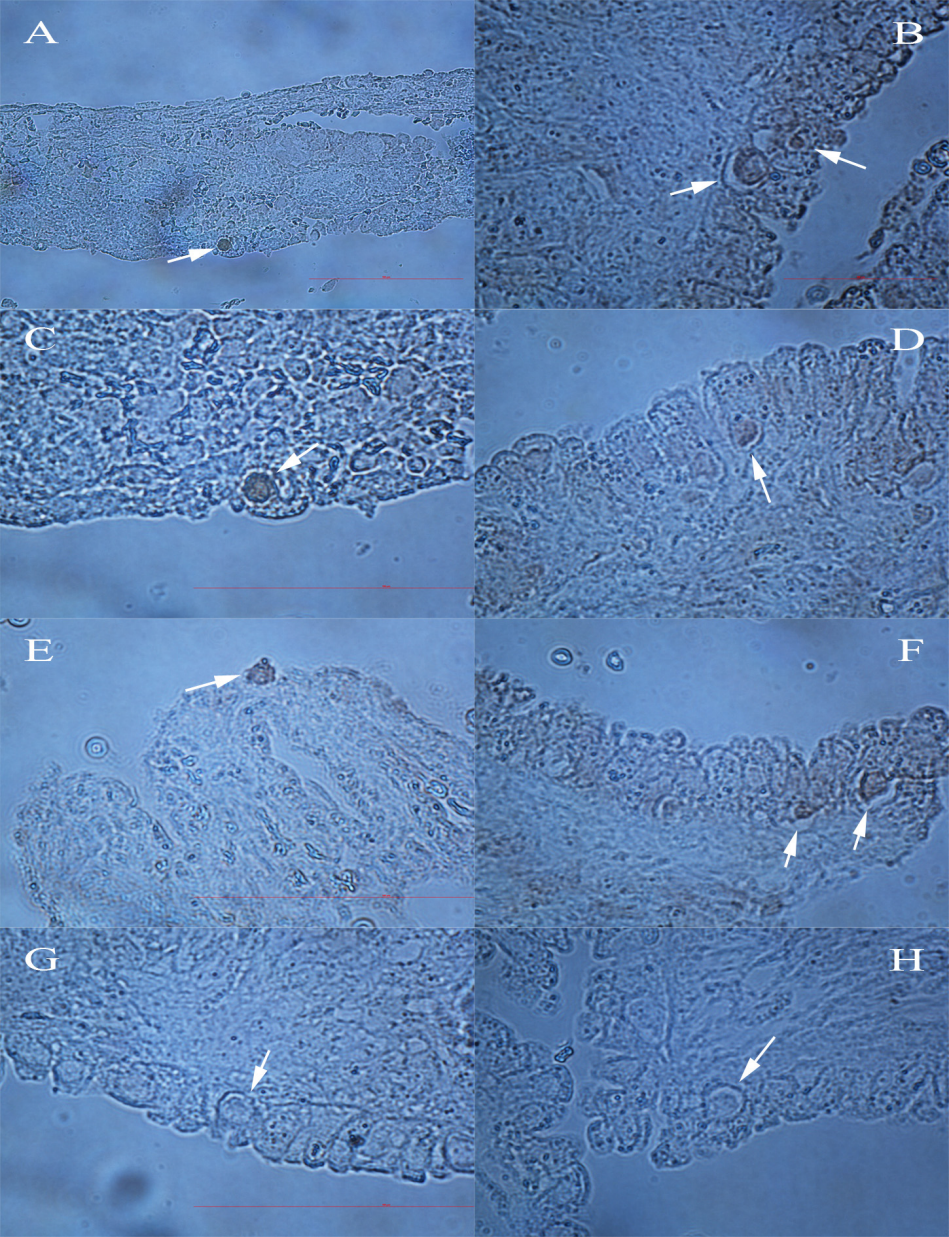
**ZP2-positive cells (arrows) in the ovarian surface epithelium**. **(A-F) **Bigger cells with diameters of approximately 10 μm. **(B, F) **Also smaller cells with diameters of approximately 5 μm. **(G, H) **Negative controls. (light microscopy, magnifications 400× and 1000×). *Legend*: brown-positive cells (Peroxidase/DAB). Scale bar: 100 μm.

Ovarian tissue was surgically retrieved from a 67-year old patient. It was paraffin embedded, cut into sections, histologically analysed, and serous papillary adenocarcinoma was diagnosed. After hematoxylin-eosin staining some *corpora albicans*, atretic follicles, small inclusion cysts, and *rete ovarii *were observed. Randomly, a proportion of ovarian cortex sections was deparaffinized and immunohistochemically stained for the expression of SSEA-4 (FITC-conjugated antibodies) and SOX-2 (PE-conjugated antibodies), the markers of pluripotency, to search for the presence of putative stem cells. The analysis confirmed the presence of round, SSEA-4 and SOX-2-positive primitive oocyte-like cells in the ovarian surface epithelium. These cells resembled primitive oocytes in the ovarian surface epithelium of fetal ovaries (Figure 1). If not primitive oocytes, these cells might be oogonia or stem cells expressing markers of pluripotency. They were round, morphologically resembled primitive oocytes with diameters from 10 to 15 μm, and were present in empty places looking like "chambers" among epithelial cells (Figures 2 and 3) quite comparable to fetal ovaries before the formation of follicles (Figure 1). Additional immunohistochemistry revealed that a proportion of these cells was positively stained on the oocyte-specific markers VASA (Figure 4) and ZP2 (Figure 5). More detailed observation revealed two populations of these cells: smaller ones with diameters of up to 5 μm (Figures 4A, B and 5B, F) resembling VSELs and bigger ones with diameters of around 10 μm (Figures 4, 5). VASA staining also confirmed the presence of rare positively stained bigger round cells with diameters of up to 30 μm in the ovarian cortex bellow the ovarian surface epithelium (Figure 4G, H). These cells were not present in follicles like normally in women of reproductive age, but were appearing as individual cells integrated in the ovarian cortical tissue.

**Figure 6:**
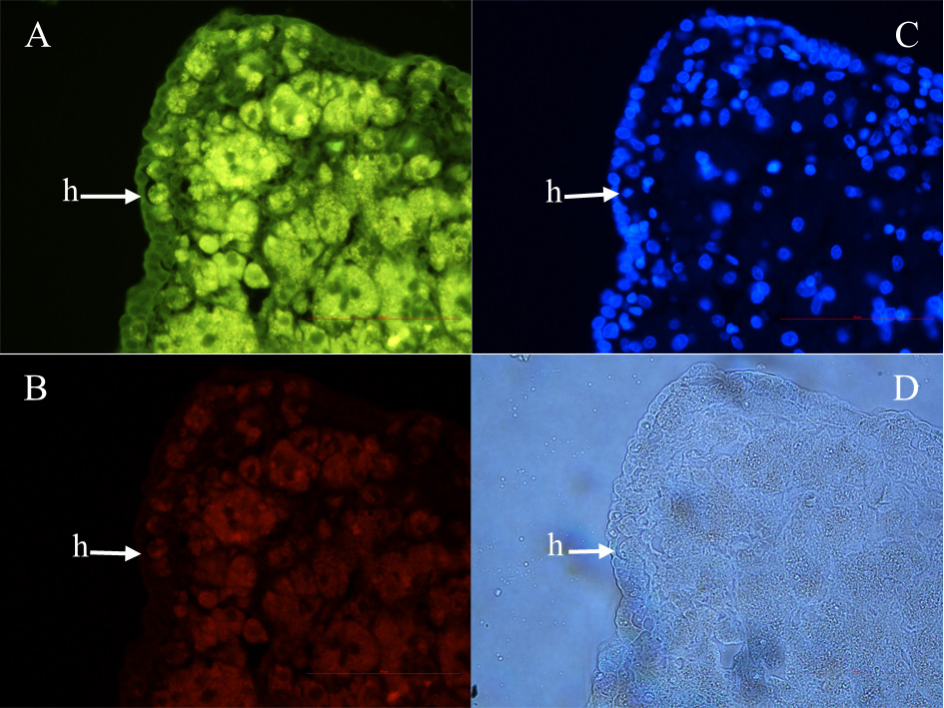
**Autofluorescent necrotic malignant tissue with clearly visible hypertrophic primitive oocyte-like cells (arrows) releasing from their "chambers"**. **(A) **Stained for the expression of SSEA-4, marker (green) of pluripotency. **(B) **Stained for the expression of SOX-2, marker (red) of pluripotency. **(C) **After DAPI (blue) staining. **(D) **Non-stained. (fluorescence and light microscopy, magnifications 400×). *Legend*: h-hypertrophic/necrotic cells.

Primitive oocyte-like cells were mostly present near the autofluorescent necrotic malignant tissue. At some places it was clearly seen that primitive oocyte-like cells were released from their "chambers" in the ovarian surface epithelium and started to change into hypertrophic/necrotic cells (Figures 3 and 6) and further into autofluorescent necrotic malignant tissue, which protruded deeper into the ovarian cortex. The nuclei of hypertrophic/necrotic cells consisted of degraded chromatin as revealed by DAPI staining (Figure 6C). This type of cells is usually not present in the healthy adult human ovarian surface epithelium.

## Discussion

Serous adenocarcinoma is a type of epithelial ovarian cancer, which is the most common among ovarian cancers. Ovarian cancers account for 6 percent of all cancers among women according to the American Cancer Society. The five-year survival rate in women with advanced ovarian cancer is 15 to 20 percent, but if the disease is found at an early stage, survival approaches 90 percent [16]. Women with a personal/family history of ovarian or other cancers are at the highest risk of having ovarian serous carcinoma, especially if their mother or sister had ovarian cancer. Other risk factors include: increased age, use of high-dose estrogens without progesterone for a long period, uninterrupted ovulation due to infertility, no pregnancies, no use of birth control, and defects in the *BRCA1 *or *BRCA2 *genes. Unfortunately, in most women ovarian serous carcinoma is not diagnosed until the disease is advanced, and has spread into the abdomen or beyond due to non-clear physical symptoms. Therefore, early diagnosis is very important. Here we report the presence of primitive oocyte-like cells in the adult human ovarian surface epithelium as related to epithelial ovarian cancer. These cells resembled primitive oocytes in the ovarian surface epithelium of fetal ovaries, and might have been involved in the manifestation of serous papillary adenocarcinoma in this patient. They expressed the analyzed markers of pluripotency SSEA-4 and SOX-2 and oocyte-specific markers VASA and ZP2 (glycoprotein of zona pellucida), therefore, the germline character of these cells is quite possible. The primitive oocyte-like cells in the ovarian surface epithelium of this patient might have persisted from the fetal period of life or developed from the putative stem cells in the ovarian surface epithelium. They might present a pathological state leading to the manifestation of ovarian serous papillary adenocarcinoma. It has been confirmed that teratoma and other germ cell tumors can be formed from oocytes/parthenogenetic embryos [17,18]. Similar primitive oocyte-like cells as reported here have already been described in the adult ovarian surface epithelium in a mouse model; ovarian surface epithelium of adult mouse ovaries seems to possess rare premeiotic germ cells that can generate oocytes following transplantation into a young host environment [19], but to our knowledge there has been no evidence in humans until now.

## Conclusion

Primitive oocyte-like cells present in the adult ovarian surface epithelium of the postmenopausal patient that probably persisted from the fetal period of life or had developed from putative stem cells in the ovarian surface epithelium are a pathological condition and might be related to serous papillary adenocarcinoma manifestation in this patient. This observation needs attention to be further investigated.

## Consent statement

Written informed consent was obtained from the patient for publication of this case report and accompanying images.

## Abbreviations

FITC: Fluorescein Isothiocyanate; PE: Phycoerythrin; SSEA-4: Stage Specific Embryonic Antigen-4; SOX-2: SRY-related HMG-box-2.

## Competing interests

The authors declare that they have no competing interests.

## Authors' contributions

IVK: performed histological analysis of ovarian sections stained for the markers of pluripotency, found the result and wrote this case report. TS: participated in the research, provided antibodies for immunohistochemistry, read and corrected the manuscript of this case report. BC: performed surgical treatment of the patient and obtained the ovarian tissue. MS: performed immunohistochemical staining of ovarian sections. JS: prepared the ovarian tissue sections to be analyzed, performed a classical histological analysis, and diagnosed the ovarian cancer. All authors read and approved the final manuscript.
